# Surface Functionalization of SBA-15 for Immobilization of Myoglobin

**DOI:** 10.3389/fbioe.2022.907855

**Published:** 2022-05-19

**Authors:** Hengmin Miao, Maosheng Li, Fang Wang, Jiao Li, Ying-Wu Lin, Jiakun Xu

**Affiliations:** ^1^ Key Laboratory of Sustainable Development of Polar Fisheries, Ministry of Agriculture and Rural Affairs, Yellow Sea Fisheries Research Institute, Chinese Academy of Fishery Sciences, Lab for Marine Drugs and Byproducts of Pilot National Lab for Marine Science and Technology, Qingdao, China; ^2^ School of Materials Science and Engineering, Shandong University of Technology, Zibo, China; ^3^ School of Food Science and Engineering, Qilu University of Technology (Shandong Academy of Sciences), Jinan, China; ^4^ School of Chemistry and Chemical Engineering, University of South China, Hengyang, China; ^5^ Key Laboratory of Protein Structure and Function of Universities in Hunan Province, University of South China, Hengyang, China

**Keywords:** myoglobin, SBA-15, organic functionalization, protein immobilization, biocatalysis

## Abstract

Mesoporous molecular sieve SBA-15 was successfully modified with 3-aminopropyltriethoxysilane (APTES) and 3-glycidyloxypropyltrimethoxysilane (GPTMS). The functionalized SBA-15 were characterized by small-angle X-ray (SAXRD), thermogravimetric analysis (TG), N_2_ adsorption, and Fourier transformed infrared spectrum (FT-IR). APTES functionalized SBA-15 (named SBA-15-A) and GPTMS functionalized SBA-15 (named SBA-15-G) were used to immobilize myoglobin (Mb). The loading amounts of Mb by SBA-15-A and SBA-15-G were 511.2 and 547.8 mg/g, respectively, whereas only 359.6 mg/g was achieved by SBA-15. Mb/SBA-15-G and Mb/SBA-15-A demonstrated better reusability than SBA-15, retaining 84.6% and 82.7% of the initial activity after repeated use seven times. The Mb/SBA-15-A and Mb/SBA-15-G also exhibited improved thermal stability and storage stability.

## 1 Introduction

The enzyme, as a natural catalyst, has the characteristics and advantages of strong catalytic selectivity, fast reaction speed, mild reaction conditions, and being clean and pollution-free. It is widely used in medicine, food, chemical industry, energy, and environmental protection, among other fields (Yu et al., 2011; Alkan et al., 2009; Mislovicová et al., 2004; Katchalski-Katzir and Kraemer, 2000). At present, industrial biotechnology, which relies on biocatalysis as its core technology, has become an important engine driving the rapid development of the social economy. As a peroxidase, myoglobin can catalyze a variety of reactions, including the oxidation of guaiacol and indole (Xu et al., 2012) and the decolorization of industrial dyes (Zhang et al., 2019a). As a result, the efficient recovery and reuse of myoglobin is the foundation of the economic and industrial application of myoglobin to improve the reusability of myoglobin; immobilizing myoglobin on a solid support is a good solution (Jakub et al., 2018; Zhao et al., 2006), which has attracted extensive attention.

To date, a variety of new types of carriers and immobilization technologies have been developed to improve the reusability of enzymes and lower the cost of enzyme biocatalysts for industrial applications. These include acrylic resins molecular sieves (Katchalski-Katzir and Kraemer, 2000), aldehyde-agarose (Guisán, 1988), gelatin (Norouzian et al., 2002), magnetic microparticles of polymers (Bozhinova et al., 2004), and layered double hydroxides (LDH) (Ren et al., 2002). Among these materials, inorganic materials have garnered increasing attention due to their unique advantages, such as easy regeneration, nontoxicity, low cost, and high stability. As inorganic support, the mesoporous molecular sieves not only have the common advantages of inorganic materials but also have adjustable pores, changeable composition, and large pore volume. They are a promising immobilized enzyme carrier that is widely used in the research of enzyme immobilization. Mesoporous silica MCM-41 was first explored to immobilize enzymes by Díaz and Jr (1996). Following that, MCM-48, SBA-15, and other types of mesoporous molecular sieves were used for enzyme immobilization. SBA-15 is a form of mesoporous silica nanoparticle with uniform pore size, long-range ordered pore structure, and a more stable structure. It has received much attention in recent years.

The immobilization method can significantly influence the reuse performance of the enzyme. The physical adsorption method has the advantages of being simple, fast, and low cost, but the force between the carrier and the enzyme is too weak, and the enzyme adsorbed into the material channel is easy to leach from the material channel in the process of reuse. For example, Serra et al. found that 30% of the adsorbed lipase was leached from the support within 2 h (Serra et al., 2008). Li et al. used SBA-15 to physically adsorb lipase. Due to the weak force between the enzyme and the support, the enzyme is leached from the pore during reuse, and only 32% of the initial activity is retained after reusing it five times (Li et al., 2009). To overcome the problem of enzyme leaching, the covalent bonding between enzyme and support is a common method for reducing enzyme leaching and improving the reuse performance of the enzyme. In the previous study, many studies showed that there are rich Si-OH groups on the surface of channels of silica materials (Kawai and Tsutsumi, 1998; Kimura et al., 1998), which can serve as the sites for the anchor of organic groups. For example, Shah et al. immobilized the penicillin acylase from SBA-15 on APTES functionalized, and the results show that immobilized enzyme rarely leaked from the carrier and has good reusability.

3-Aminopropyltriethoxysilane (APTES) and 3-glycidyloxypropyltrimethoxysilane (GPTMS) have abundant surface functional groups for surface modification, which are widely used functional reagents, and their ethoxy groups can react with hydroxyl groups on the surface of mesoporous molecular sieves and graft the amino and epoxy functional groups onto the surface of mesoporous molecular sieves. In this study, we used APTES and GPTMS to functionalize SBA-15 with amino and epoxy organic groups, as shown in Supplementary Figure S1. Small-angle X-ray (SAXRD), nitrogen adsorption-desorption experiments, Fourier transform infrared spectroscopy (FT-IR), and thermogravimetry (TG) were used to characterize the materials, which were then used to immobilize myoglobin. The amino group on SBA-15-A cannot react with the amino group on myoglobin. Glutaraldehyde needs to be added as a bridge, and the aldehyde group reacts with the amino group to connect SBA-15-A with myoglobin to realize the immobilization of myoglobin. Under the condition of immobilization, the epoxy group on SBA-15-G can react directly with the amino group on myoglobin to realize the immobilization of myoglobin. The immobilization process is shown in Supplementary Figure S2. We compared the changes in immobilized myoglobin loading of SBA-15, SBA-15-A, and SBA-15-G and studied the reuse performance of the immobilized enzymes.

## 2 Materials and Methods

### 2.1 Materials

3-Aminopropyltriethoxysilane (APTES), 3-glycidyloxypropyltrimethoxysilane (GPTMS), glutaraldehyde, and guaiacol were purchased from TCI. SBA-15 was synthesized in previous reports, and H64D/V68I Mb were expressed in *E. coli* *BL21(DE3)* cells, cultured, and purified using the procedure described previously.

### 2.2 Methods

#### 2.2.1 Preparation and Functionalization of SBA-15

The SBA-15 mesoporous materials were synthesized using a previously reported method (Zhao et al., 1998a; Zhao et al., 1998b), with P123 acting as a structure-directing agent and the TMB acting as a pore dilator agent. The functionalization of SBA-15 with 3-aminopropyltriethoxysilane (APTES) and 3-glycidyloxypropyltrimethoxysilane (GPTMS) was performed using the previously reported methods (Bourkaib et al., 2021; Lu et al., 2007). 1.0 ml of APTMS or GPTMS was added to 1.0 g of SBA-15 suspended in 50 ml of toluene and refluxed overnight under an N_2_ atmosphere. Further, the resulting solid was filtered, washed, and dried to obtain the functionalized SBA-15-A and SBA-15-G.

#### 2.2.2 Characterization of SBA-15, SBA-15-A, and SBA-15-G

Nitrogen adsorption-desorption experiments were performed using an ASAP 2460 Micromeritics System. Samples were degassed in a vacuum at 200°C for 2 h before measurement. The pore diameter distributions were calculated from desorption branches using the Barrett–Joyner–Halenda (BJH) method, and the surface areas were calculated from desorption branches using the Brunauer–Emmett–Teller (BET) method. The small-angle X-ray diffraction patterns were obtained using Cu Kα radiation on a Bruker D8 advance diffractometer (Bruker, Kontich, Belgium), and the data were collected from 0.5° to 10° in 0.5° steps. Fourier transformed infrared (FT-IR) spectra were recorded on a Shimadzu 8201 PC spectrophotometer, and the samples were prepared using the standard KBr disk method. Thermogravimetric (TG) calculations were carried out on a Perkin Elmer thermogravimetric analyzer in the air (50 ml min^−1^) at 10 K min^−1^ from 30°C to 800°C.

#### 2.2.3 Adsorption of Myoglobin Onto SBA-15

The following is the procedure of Mb immobilization with SBA-15-A. A total of 5 mg SBA-15-A was mixed with 1 ml Mb solution (3 mg/ml) and stirred in 2 ml phosphate buffer (50 mM, pH6) for 15 h at 25°C. As a crosslinking agent, 2.0 wt% glutaraldehyde was added, and the mixture was stirred for 30 min at 25°C. The amount of glutaraldehyde involved in the immobilization process of SBA-15-A was optimized by varying the volumes of glutaraldehyde used in the protocol described for enzyme crosslinking. Furthermore, the reaction mixture was centrifuged, the supernatant was removed, and the precipitate was washed thrice for 1 h with 1 ml of phosphate buffer (50 mM, pH 6). The UV absorption value of the supernatant was measured at 408 nm to determine the concentration of protein in the supernatant. The final products were identified as Mb/SBA-15-A. The procedures of Mb immobilization onto SBA-15-G and SBA-15 were carried out by a similar procedure as that for SBA-15-A without the glutaraldehyde treatment. The final products were identified as Mb/SBA-15-G and Mb/SBA-15, respectively.

#### 2.2.4 Catalytic Activity of Free Mb and Immobilized Mb

The assays comparing the catalytic activity of free Mb, Mb/SBA-15, Mb/SBA-15-A, and Mb/SBA-15-G were performed using the oxidation of guaiacol, and the product was checked by monitoring the UV absorption at 470 nm (Zhang et al., 2019b). The reaction mixture (50 mM of phosphate buffer, 1 μM of Mb, and 2.5 mM of guaiacol) was incubated for 15 min at 25°C, and the reaction was initiated by the addition of H_2_O_2_ (final concentration, 10 mM). The relative activity of an immobilized enzyme is the percentage of activity retained in the immobilized enzyme when compared to free enzyme activity.

#### 2.2.5 Operational Stability Test for Mb/SBA-15, Mb/SBA-15-A, and Mb/SBA-15-G

The influence of metal ions (Ba^2+^, Ca^2+^, Mg^2+^, Mn^2+^, and Al^3+^), organic solvents (methanol, ethanol, isopropanol, glycol, and formic acid), storage time, and temperature on Mb activity were studied to assess the stability of the immobilized protein. The reactions were carried out in phosphate buffer at a concentration of 50 mM and pH 6.

The following experiment was carried out to investigate the effect of metal ions or organic solvents on Mb activity: Mb/SBA-15, Mb/SBA-15-A, and Mb/SBA-15-G were incubated for 1 h in a buffer solution (phosphate buffer, 50 mM, pH 6) containing various metal ions (10 mM) or organic solvents (10% v/v), with a control group containing only buffer solution. Then, the enzyme activity was tested. The effect of temperature on the activity was examined by incubation for 1 h in phosphate buffer (50 mM, pH6) in a temperature range between 10°C and 60°C. The effect of storage time on Mb activity was tested by determining the remaining activity after storing the Mb in phosphate buffer (50 mM, pH 6) for different time periods of 20 days at 25°C. The activity was assayed every 5 days.

#### 2.2.6 Reusability Test for Mb/SBA-15, Mb/SBA-15-A, and Mb/SBA-15-G

After centrifugation of the reaction solution, the supernatant was tested using UV, and the precipitate was washed three times with phosphate buffer (50 mM, pH 6). The reusability of the immobilized Mb was tested for seven consecutive assays using the method described above. The remaining activity was calculated by comparing it with the first assay.

## 3 Results and Discussion

### 3.1 Structural Characteristics of the SBA-15

**FIGURE 1:**
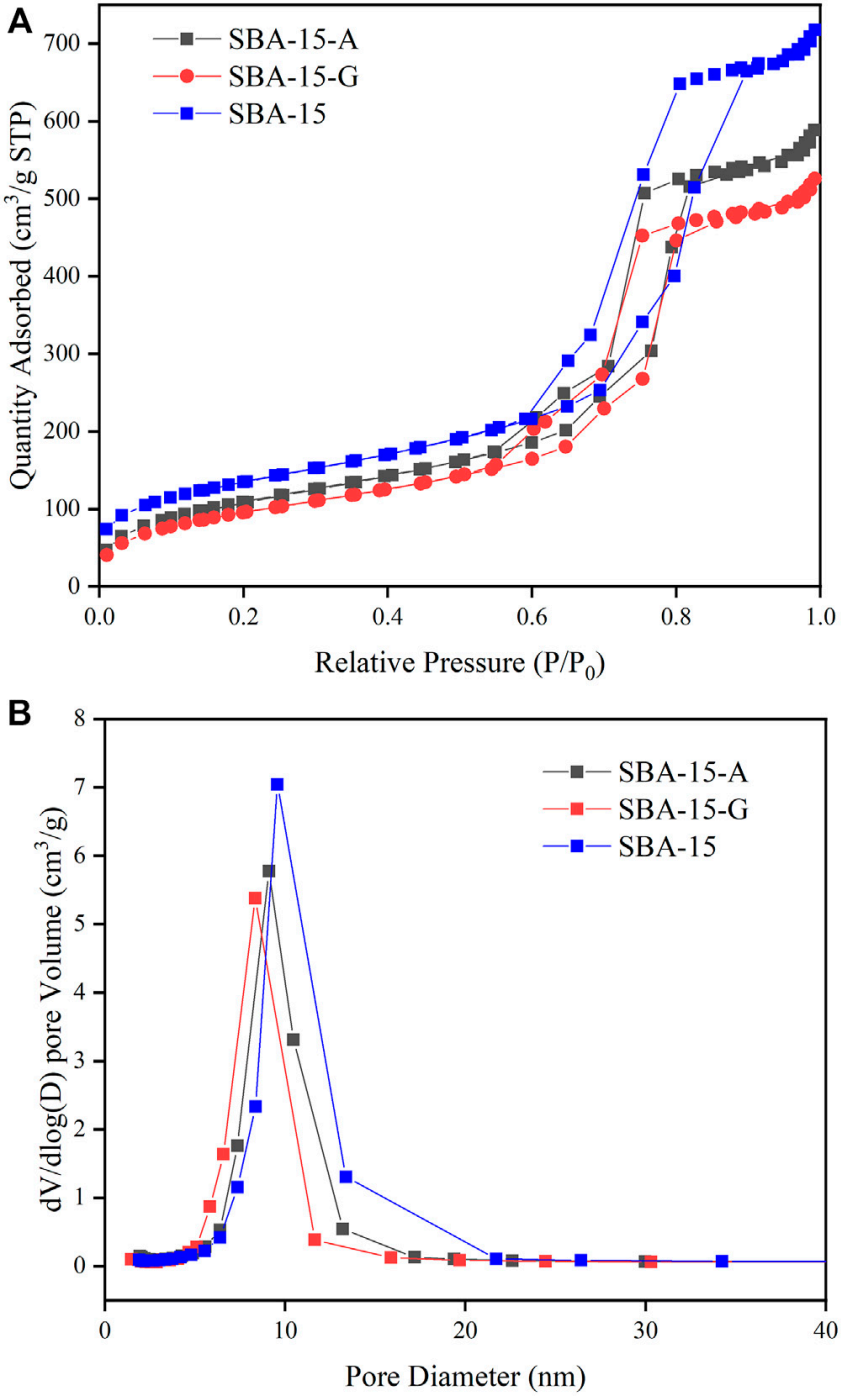
**(A)**: The adsorption isotherms of SBA-15, SBA-15-A, and SBA-15-G. **(B)**: The pore size distribution curves of SBA-15, SBA-15-A, and SBA-15-G.

The general shape of the adsorption isotherms is not modified after functionalization (Figure 1A). The adsorption curves exhibit typical Langmuir Ⅳ adsorption-desorption isotherms (Shah et al., 2008) and H1 hysteresis under high relative pressure. It belongs to hexagonal column mesoporous material. The pore size distribution curves of SBA-15, SBA-15-A, and SBA-15-G are shown in Figure 1B. From the curves, it can be seen that the pore size distribution of the modified material is reduced due to the introduction of organic groups, proving that the organic groups are successfully grafted to the surface of the material. The specific structural parameters of SBA-15, SBA-15-A, and SBA-15-G are shown in Table 1. It can be seen that the pore diameter, pore volume, and specific surface area of SBA-15-A and SBA-15-G were smaller than SBA-15, and the pore diameters of SBA-15, SBA-15-A, and SBA-15-G were 9.8, 8.7, and 8.6 nm, respectively. The decrease in pore size is due to the addition of organic groups.

**TABLE 1:** Specific structural parameters of SBA-15, SBA-15-A, and SBA-15-G.

Samples	BJH pore diameter (nm)	BET surface area (m^2^/g)	BJH pore volume (cm^3^/g)
SBA-15	9.8	484.133	1.08
SBA-15-A	8.7	434.013	0.92
SBA-15-G	8.6	403.629	0.83

Supplementary Figure S3 shows the TG diagrams of SBA-15, SBA-15-A, and SBA-15-G. The result shows that SBA-15 has a slight weight loss (3.2%) with an increase in temperature because of the evaporation of physically adsorbed water and dehydroxylation on the material surface. The functionalized samples SBA-15-A and SBA-15-G, on the contrary, have significant weight losses of 13.5% and 9.1%, respectively. The first weight loss at 25°C–100°C can be attributed to dehydration, and the latter weight loss is due to the combustion of amino groups and epoxy groups. The results of the thermogravimetric analysis in Supplementary Figure S3further showed that the amino and epoxy organic groups were successfully grafted onto the surface of SBA-15.

Small-angle XRD patterns of SBA-15, SBA-15-A, and SBA-15-G are depicted in Supplementary Figure S4. All of the samples revealed three peaks, denoted as (100), (110), and (200) reflections. These peaks are the characteristic of hexagonal mesoporous structures, associated with p6 mm hexagonal symmetry (Zhao et al., 1998a). This indicates that the long-range-order structure of the SBA-15 was not disrupted after functionalization.

**FIGURE 2:**
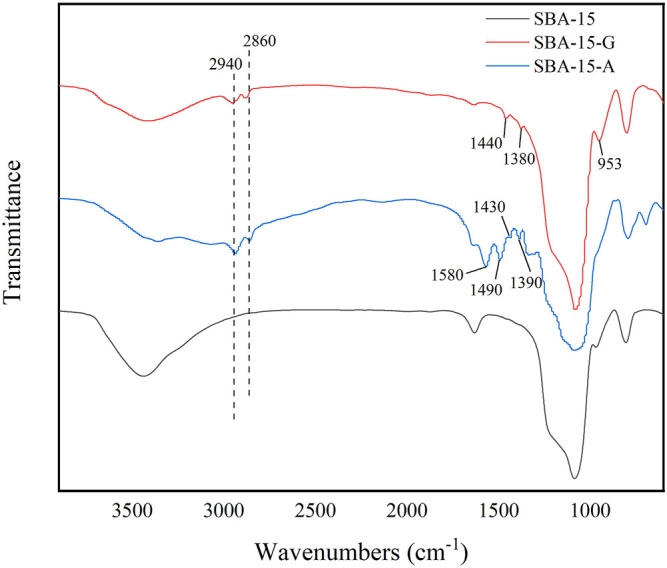
The FT-IR diagram of SBA-15, SBA-15-A, and SBA-15-G.

Figure 2 depicts the FT-IR spectra of SBA-15, SBA-15-A, and SBA-15-G. It can be seen that SBA-15 modified by amino and epoxy has a distinct absorption peak that SBA-15 does not have. In the SBA-15-A absorption spectrum, the absorption peaks near 2,940 and 2,860 cm^−1^ correspond to the symmetrical vibration and asymmetric vibration absorption of -CH_2_- (Phan and Jones, 2006), the absorption peaks at 1,580 cm^−1^ and 1,490 cm^−1^ correspond to the symmetrical bending vibration and symmetrical vibration of N-H (Nguyen et al., 2008), and the absorption peaks at 1,430 and 1,390 cm^−1^ correspond to the bending vibration absorption of C-H (Moller et al., 1999). The presence of these absorption peaks demonstrated that the amino groups were successfully grafted onto the surface of SBA-15. Similarly, in the infrared absorption spectrum of SBA-15-G, the absorption peaks near 2,940 and 2,860 cm^−1^ are asymmetric vibrations and symmetric vibration absorption of -CH_2_-, respectively; the absorption peaks at 1,440 and 1,380 cm^−1^ correspond to the bending vibration absorption of C-H; and 953 cm^−1^ corresponds to the stretching vibration of the epoxy group. These absorption peaks indicate that the epoxy group is successfully grafted to the surface of SBA-15.

### 3.2 Activity and Protein Loading Test

Table 2 compares the Mb load and retained activity of immobilized Mb in SBA-15-A and SBA-15-G mesoporous molecular sieves to SBA-15 mesoporous molecular sieves. It can be seen that SBA-15 modified by organic groups increases the load of Mb. The Mb load of SBA-15 is 359.6 mg/g, while SBA-15-G and SBA-15-A have enzyme loads of 547.8 and 511.2 mg/g, respectively, because of the protein interaction with the mesoporous material. Due to the physical adsorption of myoglobin by SBA-15, the adsorption force on the protein is small, and the protein adsorbed into the pore is easy to leak out from the pore, resulting in the low load of the material. The organic groups on the surface of the modified SBA-15 are covalently adsorbed with myoglobin adsorbed into the pore diameter. The covalent bond force is strong, making it difficult for the protein to leak out of the pore. As a result, the protein loading amount of SBA-15 was increased after modification.

**TABLE 2:** The maximum adsorption amount and remained activity of Mb onto SBA-15, SBA-15-G, and SBA-15-A.

Samples	Adsorption proteins (mg/g)	Retained activity (%)
SBA-15-G	547.8 ± 9.87	91.16 ± 1.42
SBA-15-A	511.2 ± 11.21	90.48 ± 1.46
SBA-15	359.6 ± 10.73	93.24 ± 1.36

Although the protein loading of the modified material is increased, the activity of the immobilized enzyme obtained from the modified mesoporous material is slightly reduced because the activity of the immobilized enzyme is directly related to the pore size of the mesoporous material. The addition of organic groups reduces the pore size of the carrier, which was previously conducive to the enzyme catalytic reaction (Lü et al., 2008; Miao et al., 2022). The substrate and product can diffuse well in the pore channel during the reaction process, reducing the mass transfer resistance of the substrate and product. Meanwhile, the organic group reduces the pore size of the material, and the diffusion of the substrate and product in the pore channel is affected, resulting in the reduction in the immobilized enzyme activity.

Supplementary Figure S5 shows the optimization of glutaraldehyde addition, showing the change of protein load with the additional volume of glutaraldehyde solution (2.0 wt%). It shows that the fixed load of protein is greatest when the volume of glutaraldehyde is 0.6 ml. At this time, the volume fraction of glutaraldehyde (volume ratio of protein solution to added glutaraldehyde solution) is 20%.

### 3.3 Stability of Mb/SBA-15, Mb/SBA-15-A, and Mb/SBA-15-G

**FIGURE 3:**
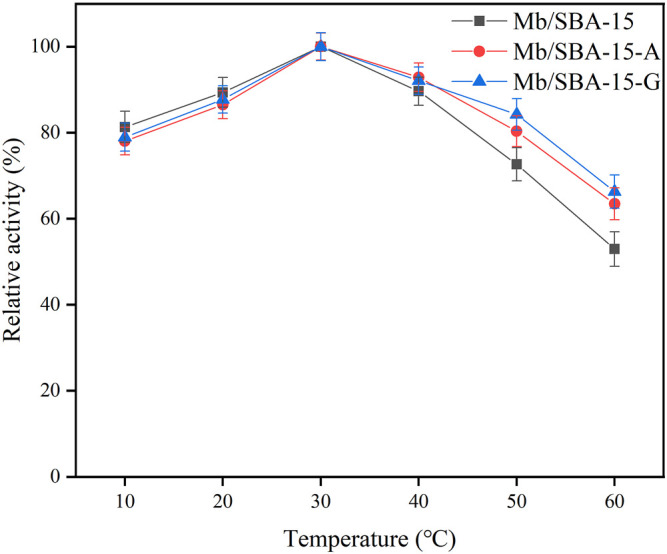
The temperature stability of immobilization Mb.

The temperature stability of the immobilized enzyme is shown in Figure 3. It shows that the optimum reaction temperature of Mb/SBA-15, Mb/SBA-15-A, and Mb/SBA-15-G was 30°C, indicating that the modification of materials does not affect the optimum reaction temperature of immobilized enzyme. We can also see that the temperature stability of the immobilized enzyme prepared by the modified material is improved. At 60°C, Mb/SBA-15 retained 52.98% of the relative enzyme activity, while Mb/SBA-15-A and Mb/SBA-15-G retained 63.48% and 66.31% of the relative enzyme activity, which were 10.5% and 13.33% higher than Mb/SBA-15, respectively, because the modified carrier was covalently connected to the enzyme molecule. This strong interaction results in the enzyme molecule having high thermal stability (Eş et al., 2015). As a result, Mb/SBA-15-A and Mb/SBA-15-G have better thermal stability than Mb/SBA-15.

**FIGURE 4:**
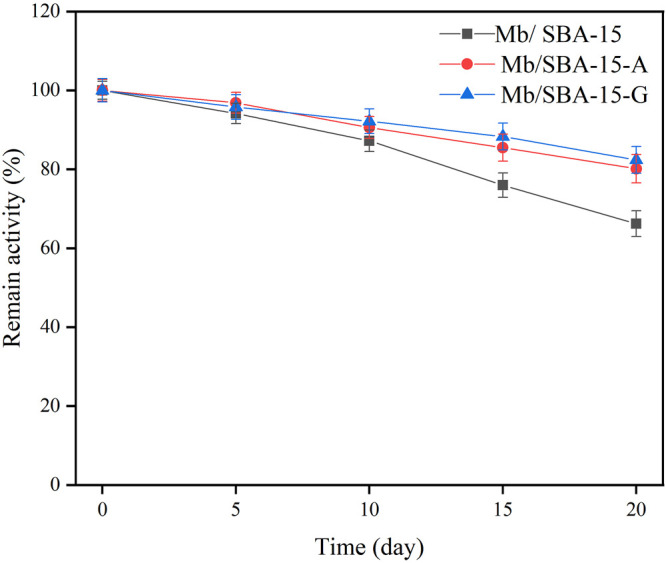
The effect of storage time on the remain activity.

The effect of storage time on the remaining activity of the immobilized enzyme is shown in Figure 4. It shows that Mb/SBA-15-A and Mb/SBA-15-G retained 80.13% and 82.41% of the remaining activity, respectively, after 20 days, whereas Mb/SBA-15 retained only 66.21%. Consequently, the Mb/SBA-15-A and Mb/SBA-15-G exhibited better storage stability than Mb/SBA-15.

Supplementary Figures S6A,B show the effects of metal ions and organic solvents on the activity of Mb/SBA-15, Mb/SBA-15-A, and Mb/SBA-15-G, respectively. It can be seen that the stability of metal ions and organic solvents of Mb/SBA-15-A and Mb/SBA-15-G were slightly lower than that of Mb/SBA-15.

### 3.4 Reusability of Mb/SBA-15, Mb/SBA-15-A, and Mb/SBA-G

**FIGURE 5:**
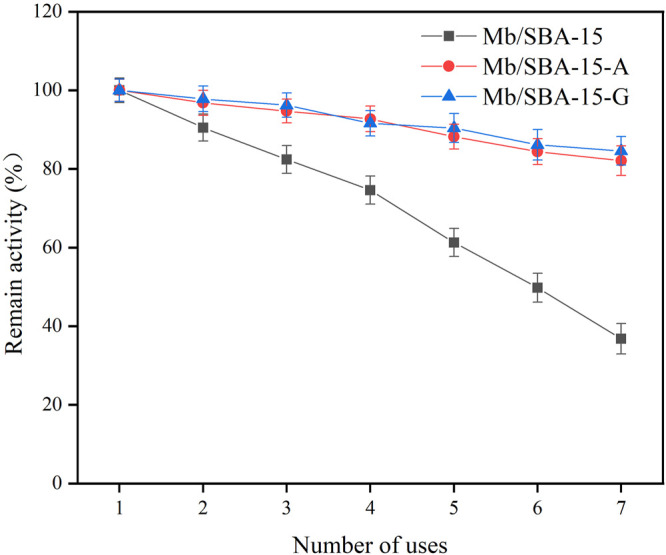
The reusability of Mb/SBA-15, Mb/SBA-15-A, and Mb/SBA-G.

The ability of immobilized enzymes to be reused is critical for their practical application. The reusability of Mb/SBA-15, Mb/SBA-15-A, and Mb/SBA-G is shown in Figure 5. The reusability of Mb/SBA-15 was poor, with only 36.41% of the initial activity being retained after seven consecutive uses. In the reuse process, the leaching of protein can be detected in the supernatant because the force between the enzyme and the carrier in Mb/SBA-15 was primarily physical adsorption, and the force is weak (Dettori et al., 2018). The enzyme will fall off from the carrier during the reuse process, resulting in a decrease in the activity of the immobilized enzyme. During the reuse of Mb/SBA-15, the cumulative enzyme leaching rate was shown in Table 3. It can be seen that after seven repeated uses, 45.12% of myoglobin was leached, which was the main reason for the decrease in protein activity. However, Mb/SBA-15-A and Mb/SBA-15-G have high reusability. Mb/SBA-15-A retains 82.7% of the initial enzyme activity, and Mb/SBA-15-G retains 84.6% of the initial enzyme activity after seven consecutive uses. In the reuse process, no protein leaching is detected in the supernatant because glutaraldehyde is used as a bridge between the enzyme and the carrier in Mb/SBA-15-A and forms a covalent bond with the amino group on the myoglobin molecule. The force between Mb/SBA-15-A immobilized enzyme myoglobin and mesoporous material is strong. Similarly, the surface of Mb/SBA-G contains an epoxy group, which can directly react with the amino group on the myoglobin molecule to form a covalent bond. The Mb/SBA-15-G immobilized enzyme has a strong force between myoglobin and mesoporous molecule. Hence, the immobilized enzyme obtained by amino and epoxy modified SBA-15 has good reusability.

**TABLE 3:** The cumulative enzyme activity loss rate of Mb/SBA-15.

Reuse times	1	2	3	4	5	6	7
Cumulative enzyme leaching rate (%)	9.21 ± 2.31	17.83 ± 2.12	25.37 ± 2.34	32.12 ± 2.11	37.55 ± 2.21	41.9 ± 2.03	45.12 ± 2.14

## 4 Conclusion

We successfully functionalized the surface of the SBA-15 mesoporous molecular sieve using APTES and GPTMS. The functionalized SBA-15 mesoporous molecular sieve was successfully grafted with organic functional groups and retained its unique pore structure. The modified SBA-15 had higher protein loading ability, higher thermal stability, better storage stability, and reuse performance compared to that of SBA-15. The improvement of the reusability of immobilized protein is of great significance for the industrial application of myoglobin.

## Data Availability

The original contributions presented in the study are included in the article/Supplementary Material, Further inquiries can be directed to the corresponding authors.
