# Supplementary material for: Surface Functionalization of SBA-15 for Immobilization of Myoglobin
Source: Front Bioeng Biotechnol. 2022 May 19;10:907855. doi: 10.3389/fbioe.2022.907855 (PMC9160787; doi:10.3389/fbioe.2022.907855)
Supplement: Supplementary file 1 [file DataSheet1.docx]

Supplementary Material

#
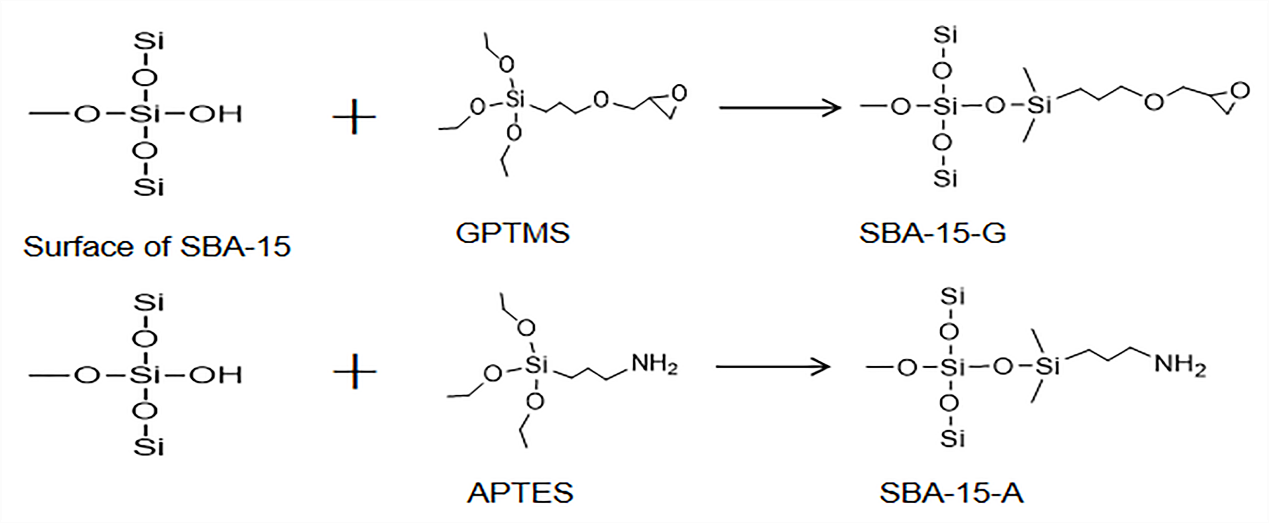
Supplementary Figures

**Supplementary Figure S1.** The mechanism for the surface functionalization of SBA-15


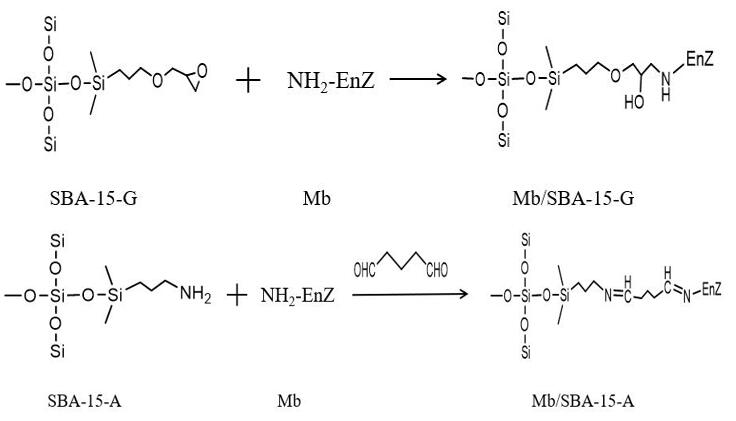


**Supplementary Figure S2.** The covalent immobilization of Mb on SBA-15-A and SBA-15-G


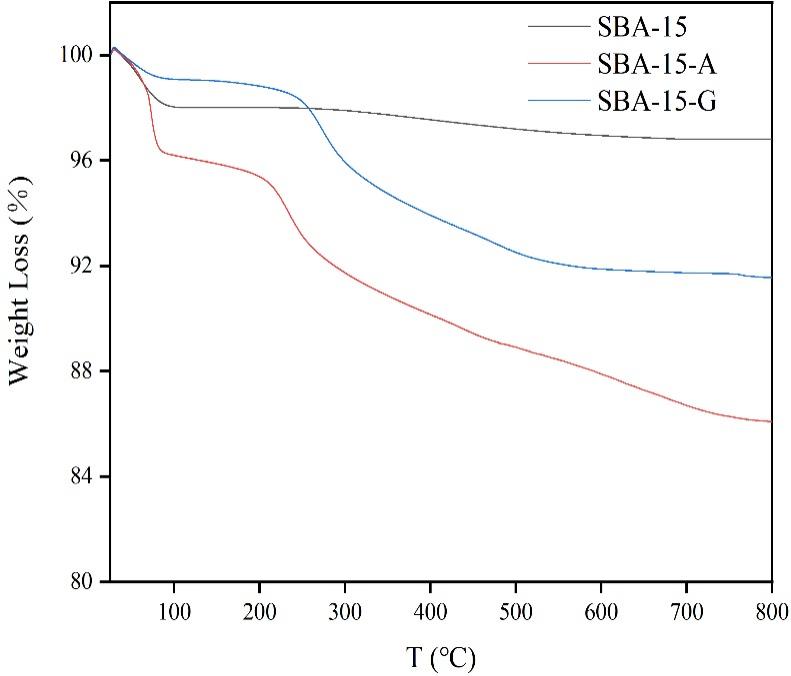


**Supplementary Figure S3.** The TG diagrams


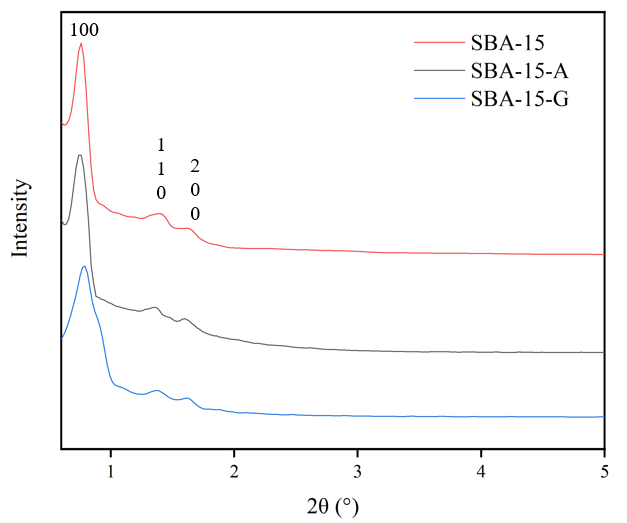


**Supplementary Figure S4.** Small-angle XRD patterns.


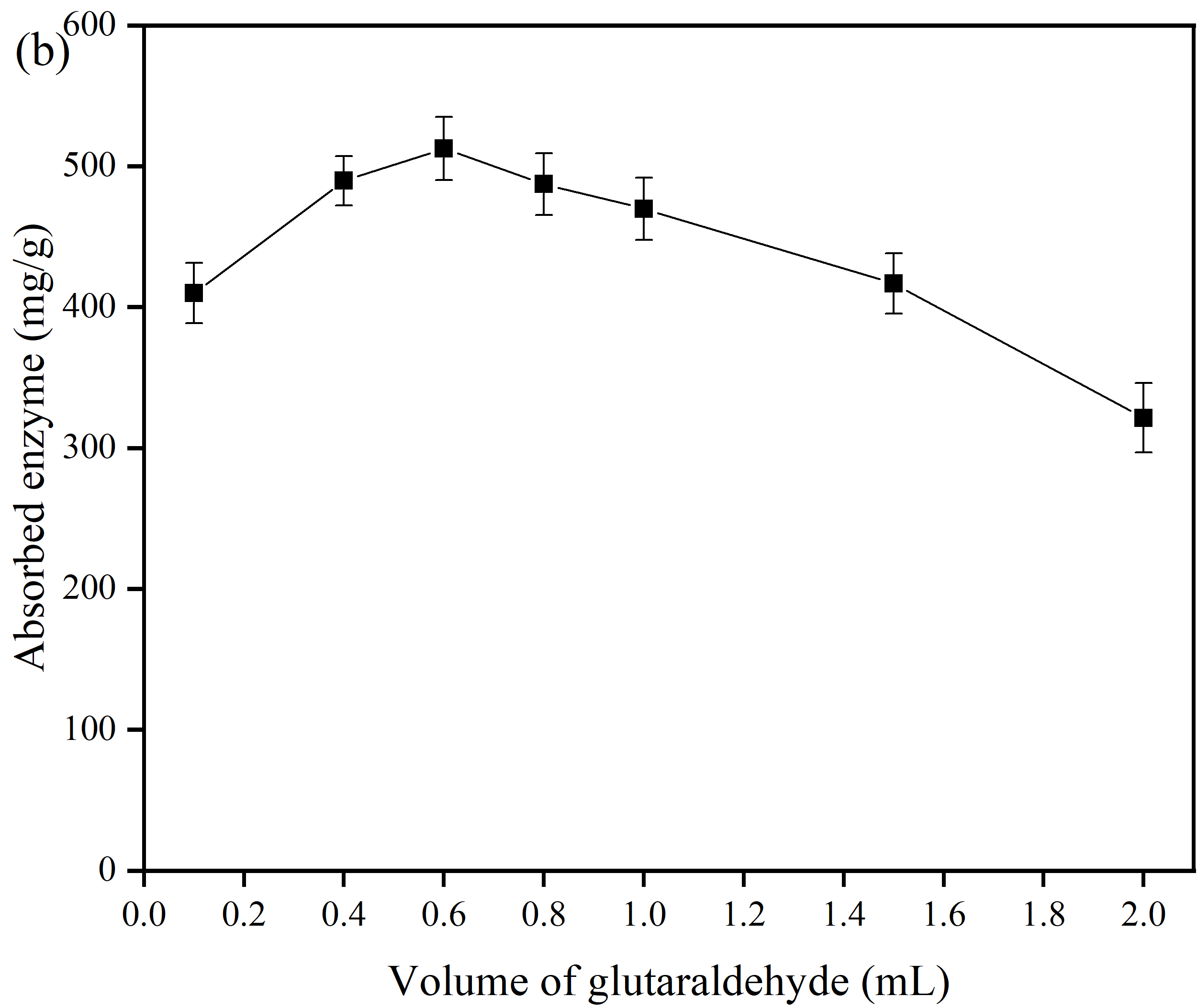


**Supplementary Figure S5.** The optimization of glutaraldehyde addition


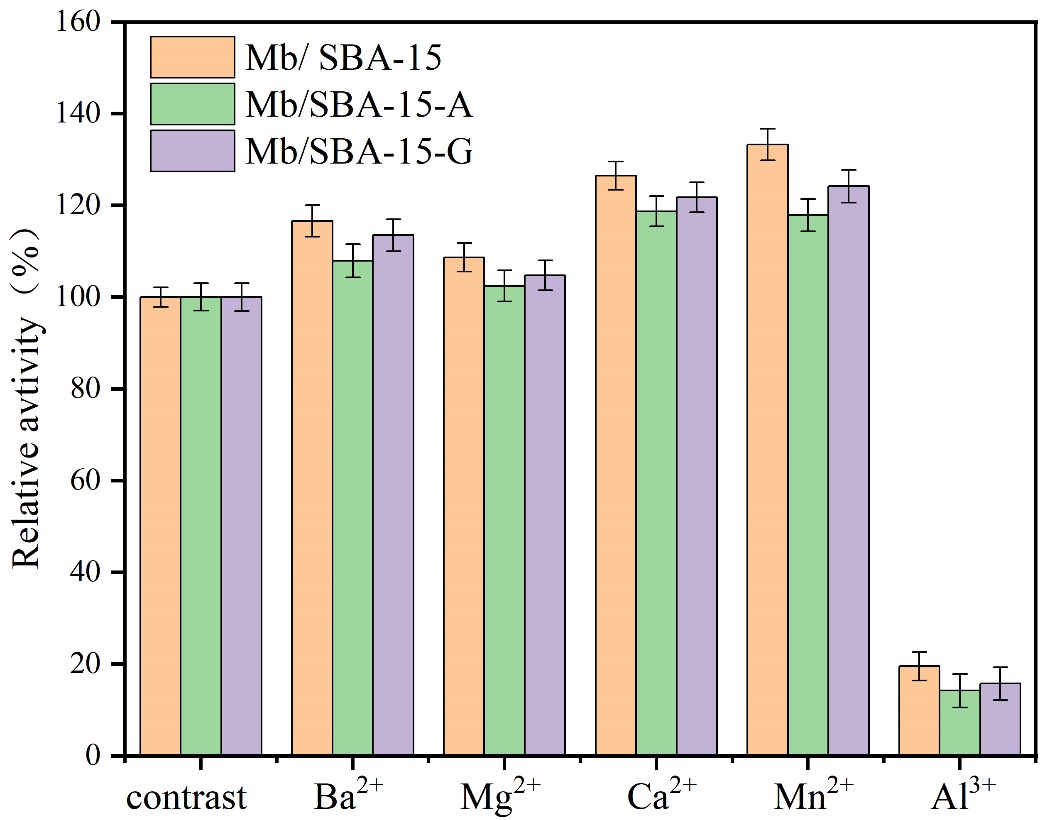


**Supplementary Figure S6 A.** The effects of metal ions.


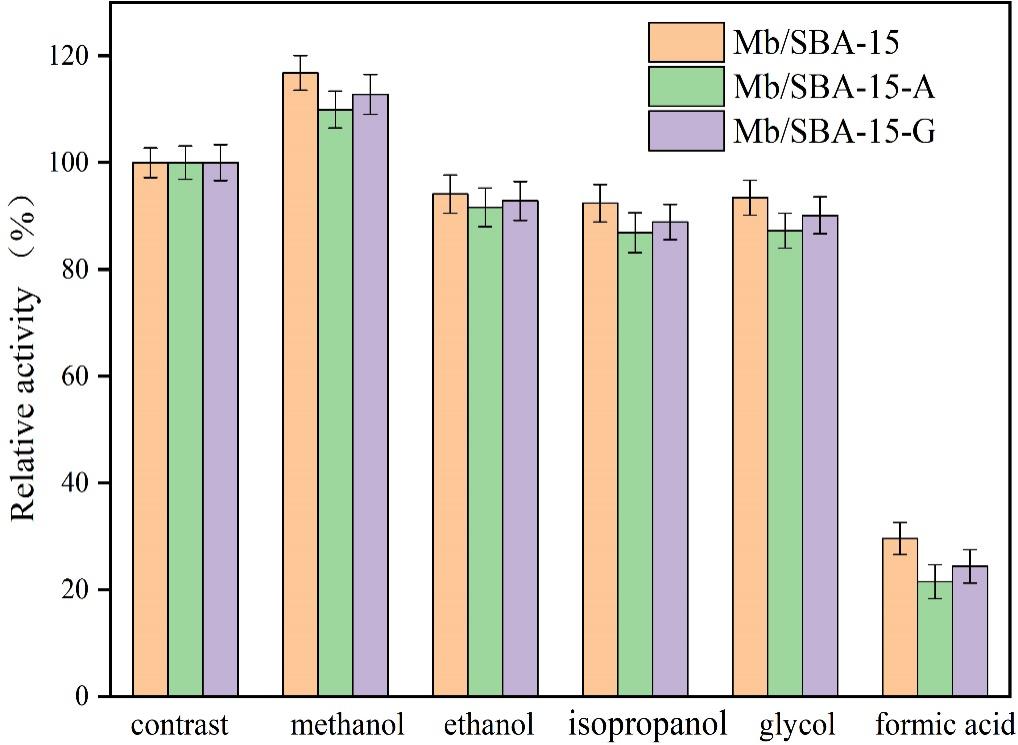


**Supplementary Figure S6 B.** The effects organic solvents.
